# Intra-Beam Interference Mitigation for the Downlink Transmission of the RIS-Assisted Hybrid Millimeter Wave System

**DOI:** 10.3390/e26030253

**Published:** 2024-03-13

**Authors:** Lou Zhao, Yuliang Zhang, Minjie Zhang, Chunshan Liu

**Affiliations:** School of Communication Engineering, Hangzhou Dianzi University, Hangzhou 310018, China; goodwin423@hdu.edu.cn (Y.Z.); zhangminjie_hdqx@163.com (M.Z.); chunshan.liu@hdu.edu.cn (C.L.)

**Keywords:** reconfigurable intelligent surface, millimeter wave, intra-beam interference, analog beamforming, low-resolution A/Ds, energy efficiency

## Abstract

Millimeter-wave (mmWave) communication systems leverage the directional beamforming capabilities of antenna arrays equipped at the base stations (BS) to counteract the inherent high propagation path loss characteristic of mmWave channels. In downlink mmWave transmissions, i.e., from the BS to users, distinguishing users within the same beam direction poses a significant challenge. Additionally, digital baseband precoding techniques are limited in their ability to mitigate inter-user interference within identical beam directions, representing a fundamental constraint in mmWave downlink transmissions. This study introduces an innovative analog beamforming-based interference mitigation strategy for downlink transmissions in reconfigurable intelligent surface (RIS)-assisted hybrid analog–digital (HAD) mmWave systems. This is achieved through the joint design of analog beamformers and the corresponding coefficients at both the RIS and the BS. We first present derived closed-form approximation expressions for the achievable rate performance in the proposed scenario and establish a stringent upper bound on this performance in a large number of RIS elements regimes. The exclusive use of analog beamforming in the downlink phase allows our proposed transmission algorithm to function efficiently when equipped with low-resolution analog-to-digital/digital-to-analog converters (A/Ds) at the BS. The energy efficiency of the downlink transmission is evaluated through the deployment of six-bit A/Ds and six-bit pulse-amplitude modulation (PAM) signals across varying numbers of activated RIS elements. Numerical simulation results validate the effectiveness of our proposed algorithms in comparison to various benchmark schemes.

## 1. Introduction

Millimeter-wave (mmWave) communications have not been extensively investigated and applied in cellular systems in past decades due to challenges including high propagation path loss, susceptibility to blockage, system complexity, hardware impairments, and elevated energy consumption [1,2,3]. The integration of massive multiple-input–multiple-output (MIMO) technologies at base stations featuring hundreds of antennas is deemed a promising approach to harness sufficient array gains and bandwidth in mmWave frequencies. This approach aims to enhance the capacity of communication systems, and has garnered increasing interest from both academic and industrial sectors [4,5,6,7].

However, the practical deployment of mmWave systems often involves a more reasonable number of antennas at the BS, which consequently may lack the spatial resolution necessary to differentiate between users or access points. Thus, mitigating interference within the same beam direction becomes a critical issue in downlink transmissions from the BS to users or pico-level access points. For instance, when users or pico-level access points are situated in the same directional path, the transmitted data streams are severely affected by intra-beam interference, which is challenging to alleviate [8]. In addition, solving this fundamental challenge with high energy efficiency imposes a further constraint. Recently, reconfigurable intelligent surfaces (RIS) have emerged as a promising solution offering a plethora of potential benefits, esuch as the ability to provide extra degrees of freedom, among others. An RIS consists of a reprogrammable planar surface with numerous low-cost and energy-efficient passive reflecting elements [8,9,10]. By modulating the phase of the incident signals, an RIS can intelligently manipulate the wireless environment through passive beamforming, thereby mitigating interference, enhancing edge coverage against blockage, and providing additional degrees of freedom by synthesizing enhanced propagation paths for transmissions [8,11].

Lately, a number of studies have explored rate performance maximization or inter-user interference minimization in RIS-assisted communication systems [9,12,13]. Unfortunately, these works employed complex fully-digital or full-duplex architectures at the BS, both of which have configurations that are overly complex and costly for practical mmWave systems. As a compromise, practical mmWave systems usually adopt hybrid analog–digital (HAD) architectures, in which a limited number of radio frequency (RF) chains can connect with a large number of antennas [6,14,15]. Thus, the proposed algorithms for maximizing the sum-rate performance while relying on a fully digital architecture cannot be applied to RIS-assisted mmWave systems with HAD architectures. Subsequent research has further investigated HAD mmWave systems assisted by RIS [16,17]. Yet, effectively addressing intra-beam inter-user interference in HAD mmWave systems assisted with RIS remains an open challenge with many research possibilities [8,15,18].

In general, the HAD architecture is equipped with high precision A/Ds, e.g., 10∼14 bits, at both transceivers to make the digital precoding design feasible for hybrid beamforming strategies. However, the utilization of high-resolution A/Ds and power amplifiers (PAs) in RF chains at transceivers is costly and dominates the total power consumption of the HAD mmWave system [15]. Thus, adopting low-resolution A/Ds at transceivers to further enhance the energy efficiency of the downlink transmission is an attractive option. This approach, however, introduces a significant challenge in mitigating intra-beam interference, as digitally quantized signals at the BS are significantly distorted, making the nonlinear quantization errors difficult to compensate for. Consequently, conventional digital precoding techniques such as zero-forcing (ZF) and minimum mean-square estimation (MMSE) fall short in effectively suppressing intra-beam co-channel interference. Thus, most algorithms designed for conventional RIS-assisted HAD mmWave systems face severe performance degradation under the impact of quantization errors due to their utilization of low-resolution A/Ds [8,9,10,16,17,18]. As such, effectively mitigating the intra-beam inter-user interference with high energy efficiency represents another critical challenge for HAD RIS-assisted mmWave systems.

To date, only a limited number of studies have investigated RIS-assisted hybrid mmWave systems with low-resolution A/Ds [18,19,20,21,22]. We listed some related works in Table 1. For instance, the authors of [18,19] sought to maximize the achievable rate of RIS-assisted mmWave systems using the particle swarm optimization (PSO) method and a block coordinated descent (BCD)-based algorithm, respectively. Moreover, in [20,21] the authors examined the achievable rate performance in RIS-aided systems under certain practical hardware imperfections, e.g., phase noise of RIS and quantization errors of A/Ds. However, these works [22,23] did not specifically take into account the intra-beam inter-user interference mitigation problem. The advantages and disadvantages of previous related works are summarized in the following comparative table to expand our main ideas.

Motivated by these considerations, this work addresses the interference mitigation challenge within the same beamforming direction for downlink transmissions in RIS-assisted HAD mmWave systems equipped with low-resolution A/Ds at the BS. The proposed algorithm jointly designs all coefficients of analog beamforming vectors at the BS and RIS to mitigate inter-user interference within the same beam direction, a problem that is not solvable in conventional HAD mmWave communication setups. This approach obviates the need for complex digital baseband precoding at the BS and any signal processing at the user. Moreover, it leverages low-resolution A/Ds at the BS to further enhance the energy efficiency of downlink transmissions. Our primary contributions are summarized as follows:

- We introduce a novel three-step non-iterative interference mitigation algorithm for designing analog-only beamformer coefficients at both the BS and the RIS, assuming perfect channel state information (CSI) at the BS. The proposed algorithm achieves comparable sum-rate performance to more computationally intensive optimization-based methods with fully digital architectures, as demonstrated in [9] within the low-to-medium signal-to-noise ratio (SNR) range, e.g., SNR∈[−15,5] dB.
- We analyze the achievable sum-rate performance of the downlink transmission within the proposed framework, deriving closed-form approximation expressions for the same. Additionally, we establish a tight upper bound on the achievable sum-rate in scenarios with a large number of RIS elements. The energy efficiency of the system is assessed across different numbers of activated RIS elements using six-bit A/Ds at the BS and six-bit pulse-amplitude modulation (PAM) signals, as suggested in [15,24].
- Numerical simulations validate the efficacy of our proposed algorithm in mitigating intra-beam interference and highlight its superior energy efficiency in downlink transmissions compared to various benchmark schemes even with a relatively limited number of RIS elements.

Notation: $E_{h}\left(\cdot \right)$ denotes a statistical expectation operation with respect to a random variable *h*; $C^{N\times M}$ denotes the space of N×M matrices with complex entries; the superscripts ${\left(\cdot \right)}^{T}$, ${\left(\cdot \right)}^{H}$, ${\left(\cdot \right)}^{*}$, and  ${\left(\cdot \right)}^{†}$ denote transpose, Hermitian transpose, conjugate, and pseudo-inverse operations, respectively; |·| denotes normal operations; the circularly symmetric complex Gaussian distribution with mean x and covariance $\sigma^{2}I$ is denoted by $\mathrm{CN}(x,\sigma^{2}I)$; ∼ means “distributed as”; and $I_{P}$ is an P×P identity matrix.

## 2. System and Channel Model

In this section, we first describe the system model adopted in this work and then detail the channel model and the downlink transmission procedure.

**Figure 1:**
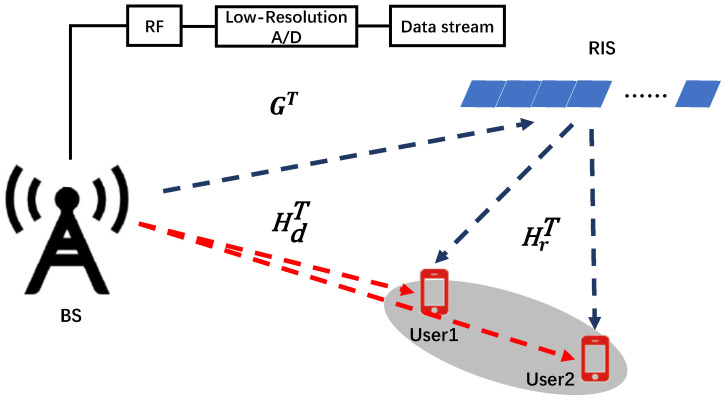
An intra-beam interference mitigation scenario of the considered multi-user RIS-assisted HAD mmWave System.

For setting up the system, we consider intra-beam interference mitigation during the downlink transmission of a multi-user RIS-assisted HAD mmWave system equipped with low-resolution A/Ds, where two independent data streams are quantized and simultaneously transmitted from the BS to two users located in the same beam direction. The BS is equipped with an *M*-antenna array, while each user is assumed to be equipped with a single omnidirectional antenna and a single RF chain. Meanwhile, the HAD BS is equipped with $N_{\mathrm{RF}}$ RF chains and an *M*-antenna uniform linear antenna (ULA) array, with each RF chain connected to *M* antennas. The system is further assisted by an *N*-element RIS (the RIS can be remotely controlled at the BS), as demonstrated in Figure 1. In addition, the two users are assumed to be located in the same beam direction of the BS at different distances. Furthermore, we assume that the users, the RIS, and the BS are fully synchronized in time and operating in time division duplex (TDD) mode.

### 2.1. Channel Model

The channels, $H_{d}^{T}\;\in \;C^{2\times M}\;=\;{\left[\;\begin{array}{c} h_{d,1},h_{d,2} \end{array}\;\right]}^{T}$, $H_{r}^{T}\;\in \;C^{2\times N}\;=\;{\left[\;\begin{array}{c} h_{r,1},h_{r,2} \end{array}\;\right]}^{T}$, and $G^{T}\in C^{N\times M}$, denote the downlink channels from the BS to the users, from the RIS to users, and from the BS to the RIS, respectively. We assume that all channels follow the block Rician fading distribution, i.e., channels are constant in a block and vary from one block to another. Specifically, $h_{d,k}^{T}$ and $h_{r,k}^{T}$, k∈{1,2} can be expressed as

(1) $$\;h_{d,k}^{T}\;=\;{\bar{\rho }}_{d}\;\left(\;\sqrt{\frac{\varsigma_{d}}{\varsigma_{d}\;+\;1}}h_{d,L,k}^{T}\;+\;\sqrt{\frac{1}{\varsigma_{d}\;+\;1}}\sqrt{\frac{1}{N_{\mathrm{cl}}}}\overset{N_{\mathrm{cl}}}{\sum_{i=1}}{\tilde{\alpha }}_{k,i}h_{d,S,k,i}^{T}\;\right)$$

and

(2) $$\;h_{r,k}^{T}\;=\;{\bar{\rho }}_{r}\;\left(\;\sqrt{\frac{\varsigma_{k}}{\varsigma_{k}\;+\;1}}h_{r,L,k}^{T}\;+\;\sqrt{\frac{1}{\varsigma_{k}\;+\;1}}\sqrt{\frac{1}{N_{\mathrm{cl}}}}\overset{N_{\mathrm{cl}}}{\sum_{i=1}}{\bar{\alpha }}_{k,i}h_{r,S,k,i}^{T}\;\right),$$

where $h_{d,L,k}\;\in \;C^{M\times 1}$ and $h_{r,L,k}\;\in \;C^{N\times 1}$ are ULA array response vectors associated with the strongest AoA components of user *k* at the BS and the RIS, which can be expressed as

(3) $$\;h_{d,L,k}\;=\;{\left[\;\begin{array}{c} 1,e^{-j\frac{2\pi d}{\lambda }\;\cos\left(\;\theta_{d,L,k}\;\right)},\cdots \;,e^{-j\frac{2\pi d}{\lambda }\left(\;M\;-\;1\;\right)\cos\left(\;\theta_{d,L,k}\;\right)} \end{array}\;\right]}^{T}\;$$

and

(4) $$\;h_{r,L,k}\;=\;{\left[\;\begin{array}{c} 1,e^{-j\frac{2\pi d}{\lambda }\cos\left(\;\theta_{r,L,k}\;\right)},\cdots \;,e^{-j\frac{2\pi d}{\lambda }\left(\;N\;-\;1\;\right)\cos\left(\;\theta_{r,L,k}\;\right)} \end{array}\;\right]}^{T}\;,$$

respectively. Variables $\theta_{d,L,k}$ and $\theta_{r,L,k}$ are the incidence AoAs from user *k* to the BS and to the RIS, ${\bar{\rho }}_{d}$ and ${\bar{\rho }}_{r}$ represent the corresponding average path loss coefficients of the BS–RIS and RIS–Users channels, and ${\tilde{\alpha }}_{k,i}$ and ${\bar{\alpha }}_{k,i}$, $i\;\in \;\{1,\cdots ,N_{\mathrm{cl}}\}$ represent the complex small-scale fading for the *i*-th scattering component of $h_{d,S,k,i}^{T}$ and $h_{r,S,k,i}^{T}$, respectively, which we assume to follow a complex Gaussian distribution with zero mean and an unit variance. Note that $\varsigma_{d}=1/\left[\sum_{i=1}^{N_{\mathrm{cl}}}{\left|{\tilde{\alpha }}_{k,i}\right|}^{2}\right]$ and $\varsigma_{r}=1/\left[\sum_{i=1}^{N_{\mathrm{cl}}}{\left|{\bar{\alpha }}_{k,i}\right|}^{2}\right]$ are the corresponding Rician K-factors. Here, we set $\frac{d}{\lambda }=\frac{1}{2}$, while *d* is the antenna interval and λ is the carrier wavelength.

The *i*-th path $h_{d,S,k,i}^{T}$ has the same form as the dominant channel component $h_{d,L,k}^{T}$, as shown in Equation (3) with the difference of angle $\theta_{d,L,k,i}$. In addition, the variable $\theta_{d,L,k,i}$ is the incidence angle of the *i*-th path at the antenna arrays of the BS. Similarly, we follow the same setting for $h_{r,S,k,i}^{T}$ and its corresponding angle $\theta_{r,L,k,i}$.

Meanwhile, the deterministic downlink line-of-sight (LOS) channel matrix $G^{T}\;\in \;C^{N\times M}$ between the BS and the RIS can be expressed as

(5) $$G^{T}=h_{N}^{*}\left(\varphi_{g}\right)h_{M}^{T}\left(\theta_{g}\right),$$

where the array response vectors $h_{M}\left(\cdot \right)$ and $h_{N}\left(\cdot \right)$ follow the same setting as shown in Equations (3) and (4) with the difference of angles $\varphi_{g}$ and $\theta_{g}$, respectively.

### 2.2. Transmission Procedure

For the considered downlink transmission of the RIS-assisted HAD mmWave system, the signals received at the users are $y\in C^{K\times 1}$, K=2, which can be represented as

(6) $$y\;=\;\left[H_{d}^{T}+H_{r}^{T}\Phi G^{T}\right]F_{\mathrm{RF}}F_{\mathrm{BB}}\;x\;+\;z,$$

where $x\in C^{K\times 1}$ are symbols transmitted from the BS to the users, $E\left[xx^{H}\right]=E_{s}I_{K}$, $E_{s}$ is the average symbol energy for each user, $F_{\mathrm{RF}}\in C^{M\times K}$ is the analog beamformer, $F_{\mathrm{BB}}\in C^{K\times K}$ is the digital precoder, $\Phi \in C^{N\times N}$ is the phase-coefficient matrix of the RIS, and $z\in C^{K\times 1}∼\mathrm{CN}\left(0,\sigma^{2}I\right)$ is complex white Gaussian noise with variance $\sigma^{2}$. In general, to maximize the achievable sum-rate of the considered scenario as shown in Equation (6), the optimization problem can be formulated as follows:

(7) $$\begin{array}{cc} \; & \max_{\Phi ,F_{\mathrm{RF}},Q_{\mathrm{BS}}\left(\cdot \right),p\left(\cdot \right)}I\left(x;y|\left[H_{d}^{T}+H_{r}^{T}\Phi G\right]\right)\\ \; & s.t.\;\;\;\;\;\;|F_{\mathrm{RF},i,j}|=1,\;\forall i,j,\\ \; & \;\;\;\;\;\;\;\;\;\;\;|\Phi_{i,j}|=1,\;\forall i,j,\\ \; & \;\;\;\;\;\;\;\;\;\;\;{∥F_{\mathrm{BB}}∥}_{F}^{2}=K,\\ \; & \;\;\;\;\;\;\;\;\;\;\;{∥x∥}_{F}^{2}=KE_{s}⩽P_{t},\\ \; & \;\;\;\;\;\;\;\;\;\;\;\;b<+\infty , \end{array}$$

where $p\left(x\right)$ represents the probability distribution of x and $I\left(x;y|\left[H_{d}^{T}+H_{r}^{T}\Phi G^{T}\right]\right)$ is the mutual information between two random variables x and y given a downlink channel $\left[H_{d}^{T}+H_{r}^{T}\Phi G^{T}\right]$. In general, the analog beamformer matrix for the BS is $F_{\mathrm{RF}}$, and can be expressed as

(8) $$F_{\mathrm{RF}}=\left[\begin{array}{c} f_{\mathrm{RF},1},\cdots ,f_{\mathrm{RF},K} \end{array}\right],$$

where the column vector $f_{k}$ represents the analog beamformer for user *k*. In addition, the phase-coefficient matrix of the RIS is $\Phi \in C^{N\times N}$, which can be expressed by

(9) $$\Phi =\mathrm{diag}\left[\begin{array}{c} e^{-j\phi_{1}},\cdots ,e^{-j\phi_{i}},\cdots ,e^{-j\phi_{N}} \end{array}\right],$$

where $\phi_{i}\in \left[0,+2\pi \right)$, i={1,⋯,N} denotes the reflection phase coefficient of the *i*-th RIS element. It is very nontrivial to solve such a joint optimization problem, which is highly combinatorial and intractable. Unfortunately, the problem formulated in Equation (7) is an NP-hard nonconvex problem, meaning that it cannot be solved [6,14,22].

To avoid the impact on the receive SINR performance due to quantization errors contributed by utilizing low-resolution A/Ds at the BS, we jointly utilize the analog beamformers at the BS and the RIS to synthesize cascaded channels in order to mitigate the considered interference and shift the signal processing from the digital baseband to the analog domain, i.e., $F_{\mathrm{BB}}=I$.

Thus, to evaluate the intra-beam interference, the signals received at user *k* can be rewritten as follows:

(10) $$\begin{array}{c} y_{k}=\underset{\mathrm{Desired}\;\mathrm{signal}}{\underset{︸}{\left[h_{d,k}^{T}+h_{r,k}^{T}\Phi G^{T}\right]f_{\mathrm{RF},k}x_{k}}}+\underset{\mathrm{inter}-\mathrm{user}\;\mathrm{interference}\;I_{k}}{\underset{︸}{\left[h_{d,k}^{T}+h_{r,k}^{T}\Phi G^{T}\right]\sum_{i\neq j}^{K}f_{\mathrm{RF},j}x_{j}}}+\underset{\mathrm{noise}}{\underset{︸}{z_{k}}}. \end{array}$$

It is notable that for the considered intra-beam interference mitigation scenario, users 1 and 2 have the same strongest AoA components, i.e., $h_{d,L,1}\approx h_{d,L,2}$. In conventional HAD mmWave systems, the design of analog beamforming relies on the strongest AoA components, i.e., $f_{\mathrm{RF},1}\approx f_{\mathrm{RF},2}$. Thus, during the downlink transmission, the signals desired at user 1 will cause sever inter-user interference at user 2 and vice versa. Accordingly, the receive SINR performance for user *k*, k∈{1,2} can be expressed as

(11) $$\begin{array}{c} {\mathrm{SINR}}_{k}=\frac{1}{1+\frac{\sigma^{2}}{E_{s}\left[h_{d,k}^{T}+h_{r,k}^{T}\Phi G^{T}\right]f_{\mathrm{RF},k}f_{\mathrm{RF},k}^{H}{\left[h_{d,k}^{T}+h_{r,k}^{T}\Phi G^{T}\right]}^{H}}}, \end{array}$$

where the SINR performance is upper bounded and less than 1. Then, the energy efficiency of the considered scenario is poor due to the low SINR performance.

## 3. RIS-Assisted Intra-Beam Interference Mitigation

In this section, we propose a three-step scheme to mitigate the intra-beam interference in the RIS-assisted HAD mmWave system. We evaluate and obtain the closed-form approximation expressions for the achievable rate performance. In addition, we further derive an upper bound of the achievable rate performance when the number of RIS elements is relatively large. We note here that perfect CSIs are assumed to be known at the BS.

We first detail the proposed three-step algorithm, as listed in Algorithm 1. In the first step, we adopt the multi-beam technology, i.e., the beam-splitting method, to design analog beamformers at the BS for two users. In the second step, we design the coefficients of RIS elements, which is facilitated by utilizing the CSIs of the RIS–user channels known at the BS. Then, other coefficients required for the downlink transmission, e.g., power allocation coefficients, can be designed in Step 3.

First, we adopt the beam-splitting method [25] to design analog beamformers for users 1 and 2 at the BS, provided by

(12) $$f_{\mathrm{RF},1}=\underset{\mathrm{Directional}\;\mathrm{transmission}}{\underset{︸}{\sqrt{\rho_{1}}f_{L,1}}}+\sqrt{1-\rho_{1}}f_{R,1}\;\;\mathrm{and}\;$$

(13) $$f_{\mathrm{RF},2}=\underset{\mathrm{Directional}\;\mathrm{transmission}}{\underset{︸}{\sqrt{\rho_{2}}f_{L,2}}}+\sqrt{1-\rho_{2}}f_{R,2},$$

where the analog beamformers designed for users at the BS contain two parts: the directional transmission part $f_{L}$, and the reflection transmission part $f_{R}$, as shown in Equations (12) and (13). We note here that the power allocation coefficients $\rho_{1}$, $\rho_{2}$ and analog beamformer $f_{R,1}$ for user 1 are designed in Step 3. From Equations (10) and (13), interference signals transmitted for user 2 but received at user 1 can be expressed as

(14) $$\begin{array}{c} I_{1}=\left[\sqrt{\rho_{2}}h_{d,1}^{T}f_{L,2}+\sqrt{\rho_{2}}h_{r,1}^{T}\Phi G^{T}f_{L,2}+\sqrt{1-\rho_{2}}h_{d,1}^{T}f_{R,2}+\sqrt{1-\rho_{2}}h_{r,1}^{T}\Phi G^{T}f_{R,2}\right]x_{2}. \end{array}$$

**Algorithm 1** Analog Beamforming Design for Intra-beam Interference Mitigation
**Require:** $\theta_{d,L,1}=\theta_{d,L,2}$, strongest AoAs of users at the BS, $\left[h_{r,1},h_{r,2}\right]$, CSIs of users at the RIS, and incidence angles $\varphi_{g}$ and $\theta_{g}$ between the BS and the RIS**Output:** (1) $f_{1}=\sqrt{\rho_{1}}f_{L,1}+\sqrt{1-\rho_{1}}f_{R,1}$
      (2) $f_{2}=\sqrt{\rho_{2}}f_{L,2}+\sqrt{1-\rho_{2}}f_{R,2}$      (3) Φ   STEP 1: Beam splitting algorithm  1: Design beam-splitting analog beamforming vectors for users 1: $f_{L,1}=\sqrt{\frac{1}{M}}h_{d,L,1}^{*}\left(\theta_{d,L,1}\right)$ and users 2: $f_{L,2}=\sqrt{\frac{1}{M}}h_{d,L,2}^{*}\left(\theta_{d,L,2}\right)$ and $f_{R,2}=\sqrt{\frac{1}{M}}h_{M}^{*}\left(\theta_{g}\right)$   STEP 2: Design phase shifters Φ at the RIS  2: Design phase shifters for the RIS to mitigate the dominate interference:   $\Phi ={\left(h_{r,1}^{T}⊙h_{N}^{H}\right)}^{H}e^{j\pi }$     STEP 3: Obtain power allocations coefficients  3: Obtain power allocation factor for user 2:
   $\rho_{2}=\frac{\alpha_{2}}{1+\alpha_{2}}$, where $\alpha_{2}=\frac{N^{2}\left(1+\varsigma_{k}\right)}{\varsigma_{k}}$  4: Design analog beamformer $f_{R,1}$, and obtain power allocation factor for user 1:   $f_{R,1}=\frac{1}{\sqrt{M}}h_{M}^{*}\frac{h_{r,1}^{T}h_{r,2}^{*}}{|h_{r,1}^{T}h_{r,2}^{*}|}$, and  $\rho_{1}=\frac{\alpha_{1}}{1+\alpha_{1}}$, where $\alpha_{1}=\frac{|h_{r,2}^{T}h_{r,1}^{H}{|}^{2}}{\frac{\varsigma_{k}}{1+\varsigma_{k}}}$


### Details of Intra-Beam Interference Mitigation Algorithm

Aiming to fully exploit the passive beamforming gain provided by the BS and the RIS, we now intuitively design analog beamformers to simplify the initial setting, i.e., $f_{L,2}=\sqrt{\frac{1}{M}}h_{d,L,2}^{*}\left(\theta_{d,L,2}\right)$, $f_{R,2}=\sqrt{\frac{1}{M}}h_{M}^{*}\left(\theta_{g}\right)$, and $f_{L,1}=\sqrt{\frac{1}{M}}h_{d,L,1}^{*}\left(\theta_{d,L,1}\right)$. Then, we focus on designing Φ to reflect signals for mitigating the dominate intra-beam interference, i.e.,

(15) $$\begin{array}{c} \sqrt{\rho_{2}}h_{d,1}^{T}f_{L,2}=-\sqrt{1-\rho_{2}}h_{r,1}^{T}\Phi G^{T}f_{R,2}. \end{array}$$

Thus, we can further rewrite Equation (15) as

(16) $$\begin{array}{c} -\sqrt{\frac{\rho_{2}\varsigma_{k}}{\left(1-\rho_{2}\right)\left(1+\varsigma_{k}\right)}}=h_{r,1}^{T}\Phi h_{N}^{*}=h_{r,1}^{T}⊙h_{N}^{H}\varphi , \end{array}$$

where $\Phi =\mathrm{diag}\left[\varphi \right]$. Consequently, we can obtain the coefficients designed for the RIS, which are provided by

(17) $$\begin{array}{c} \varphi_{D}={\left(h_{r,1}^{T}⊙h_{N}^{H}\right)}^{H}e^{j\pi }, \end{array}$$

and the power normalization coefficient designed for beam splitting in Equation (13), $\rho_{2}$, provided by

(18) $$\begin{array}{c} \rho_{2}=\frac{N^{2}}{\frac{\varsigma_{k}}{1+\varsigma_{k}}+N^{2}}. \end{array}$$

Similarly, we can design the analog beamformer $f_{R,1}$ following the same design principle as for user 2, which satisfies the following equation:

(19) $$\begin{array}{cc} \;\sqrt{\;\frac{\rho_{1}\varsigma_{k}}{\left(1+\varsigma_{k}\right)\;\left(1-\rho_{1}\right)}}\sqrt{M}\; & =\;h_{r,2}^{T}h_{r,1}^{*}h_{M}^{T}f_{R,1}. \end{array}$$

Thus, the reflection part of the designed analog beamformer for user 1, denoted $f_{R,1}$, can be designed to fully exploit the power gain of the RIS, which is provided by

(20) $$f_{R},1=\frac{1}{\sqrt{M}}h_{M}^{*}\frac{h_{r,1}^{T}h_{r,2}^{*}}{|h_{r,1}^{T}h_{r,2}^{*}|}.$$

In addition, we can obtain the power allocation coefficient for user 1, denoted $\alpha_{1}$, which can be expressed as

(21) $$\rho_{1}=\frac{\alpha_{1}}{1+\alpha_{1}},$$

where $\alpha_{1}={\left|h_{r,2}^{T}h_{r,1}^{*}\right|}^{2}/\left(\frac{\varsigma_{k}}{1+\varsigma_{k}}\right)$.

Now, exploiting the analog beamformers in Algorithm 1, we summarize the closed-form approximation of the achievable sum-rate in the following theorem.

**Theorem** **1.** *With the designed analog beamformers and power allocation coefficients in Algorithm 1, the achievable sum-rate performance of the considered scheme can be approximated by*

(22) $$\begin{array}{c} \hat{R}\approx \log_{2}\left[1+\frac{D_{1}}{I_{1}+\frac{\sigma^{2}}{E_{s}}}\right]+\log_{2}\left[1+\frac{D_{2}}{I_{2}+\frac{\sigma^{2}}{E_{s}}}\right], \end{array}$$

*where $D_{1}$, $D_{2}$, $I_{1}$, and $I_{2}$ are provided in Equations (A2)–(A6) in Appendix A.*

**Proof.**  Please refer to Appendix A. □

The analytical results provided in Equation (22) indicate that our proposed intra-beam interference mitigation frame can work effectively while the two users have a distinguishable angular separation from the observation frame of the RIS.

**Corollary** **1.** *In regimes with a large number of RIS elements, i.e., N→∞, the average achievable sum-rate performance can be asymptotically upper bounded by*

(23) $$\begin{array}{cc} \;\hat{R}= & \log_{2}\left[1+E\left[{\mathrm{SINR}}_{1}\right]\right]+\log_{2}\left[1+E\left[{\mathrm{SINR}}_{2}\right]\right]\\ \overset{N\to \infty }{⩽} & \log_{2}\left[1+\frac{M}{\;\frac{1}{M}\frac{\varsigma_{d}}{\varsigma_{d}+1}{\left|h_{M}^{T}h_{d,L,2}^{*}\right|}^{2}}\right]+\log_{2}\left[1+\frac{ME_{s}}{\;\frac{1}{M}{\left|h_{M}^{T}h_{d,L,1}^{*}\right|}^{2}+\sigma^{2}}\right].\; \end{array}$$

**Proof.**  By substituting $\frac{1}{N}H_{r}^{H}H_{r}\underset{N\to \infty }{\overset{a.s.}{\to }}I_{2}$ (the same method as provided in Equation (48), Corollary 4 of reference [6]) into (10), (18) and (21), we have $\rho_{1}\to 0$ and $\rho_{2}\to 1$. Then, the corresponding result is obtained after some mathematical manipulations. This completes the proof. □

From Equations (22) and (23), it can be seen that the upper bound of the achievable sum-rate of the proposed scheme is mainly determined by channel correlations between the strongest AoA components from the BS to the users ($h_{d,L,i}$, i∈{1,2}) and the strongest AoA component from the BS to the RIS ($h_{M}$). This indicates that the deployment of the RIS may have effects on the achievable rate performance.

## 4. Energy Efficiency Performance Analysis

In this section, we evaluate the energy efficiency of the considered RIS-assisted HAD architecture when the BS is equipped with six-bit A/Ds and adopts six-bit PAM signals, as suggested in [15,24,26].

Based on the receive SNRs of users 1 and 2 derived in the previous section, the achievable rate of user *k* in the considered scenario which adopts six-bit PAM signals and equipped with six-bit A/Ds at the BS can be approximated by following the methods proposed in [15]:

(24) $$\begin{array}{cc} \; & {\hat{R}}_{k}^{6-\mathrm{bit}}\approx 2\left[6-\frac{1}{2^{6}}\times \sum_{t=0}^{2^{6}-1}E_{{\tilde{w}}_{k}}\left\{\log_{2}\sum_{i=0}^{2^{6}-1}\exp\left(-\frac{|a^{t}+{\tilde{w}}_{k}-a^{i}{|}^{2}-{\left|{\tilde{w}}_{k}\right|}^{2}}{2\xi_{k}^{2}}\right)\right\}\right], \end{array}$$

where $a^{i}$ is the *i*-th element of $S_{6\mathrm{bit}-\mathrm{PAM}}$, $i\in \{1,\cdots ,2^{6}\}$, and ${\tilde{w}}_{k}∼N\left(0,\xi_{k}^{2}\right)$ with $\xi_{k}^{2}=\frac{I_{k}+\sigma^{2}/E_{s}}{2D_{k}}$. In addition, each dimension of the transmitted signals $x\in S_{6\mathrm{bit}-\mathrm{PAM}}$ is modulated by the equiprobable and equispaced six-bit PAM input signalling, which is provided by

(25) $$\begin{array}{cc} \; & S_{6\mathrm{bit}\;-\;\mathrm{PAM}}=\left\{-\frac{\left(2^{6}\;-\;1\right)\Delta }{2},-\frac{\left(2^{6}\;-\;3\right)\Delta }{2},\cdots ,\frac{\left(2^{6}\;-\;3\right)\Delta }{2},\frac{\left(2^{6}\;-\;1\right)\Delta }{2}\right\}, \end{array}$$

where Δ is the stepsize and the average symbol energy of the six-bit PAM signals is $E\left[x^{2}\right]=E_{s}$. For the considered RIS-assisted HAD mmWave system with six-bit A/Ds, we can quantify the total consumed power of the system during the downlink transmission as [15,24,27]

(26) $$\begin{array}{c} \;P_{\mathrm{BS}}\;=\;2\left[2P_{A/D}\;+\;MP_{\mathrm{PS}}\;+\;P_{\mathrm{RFC}}\;+\;P_{\mathrm{PA}}\;\right]+\;P_{t}\;+\;P_{\mathrm{BB}}\;+\;NP_{\mathrm{RIS}}, \end{array}$$

where $P_{t}$ is the total transmission power, $P_{\mathrm{BB}}$ is the baseband signal processing power consumption, $P_{\mathrm{PS}}$ is the power consumption of phase shifters, $P_{\mathrm{PA}}$ is the power consumption of the amplifier, $P_{\mathrm{RIS}}$ is the power consumption of each RIS element, and $P_{\mathrm{RFC}}$ is the power consumption of the RF chain. For *b*-bit quantization of the A/D power consumption, it can be expressed as

(27) $$P_{A/D}={\mathrm{FOM}}_{W}\cdot f_{s}\cdot 2^{b},$$

where ${\mathrm{FOM}}_{W}$ is Walden’s figure-of-merit for evaluating the A/D power efficiency with resolution and speed, $f_{s}=4W$ is the the sampling rate, *W* is the bandwidth, and *b* is the number of resolution bits. In general, the exact value of ${\mathrm{FOM}}_{W}$ depends on the design of the A/Ds and $P_{A/D}$ dominates the total power consumption $P_{\mathrm{BS}}$ when the number of quantization bits *b* is large.

Then, we can obtain the energy efficiency of the BS with six-bit PAM signals as $\eta_{\mathrm{BS}}$, which can be expressed as follows:

(28) $$\begin{array}{cc} \;\eta_{\mathrm{BS}} & =\frac{\sum_{k=1}^{2}{\hat{R}}_{k}^{6-\mathrm{bit}}}{P_{\mathrm{BS}}}\\ \; & =\frac{\sum_{k=1}^{2}2\left[6-\frac{1}{2^{6}}\times \sum_{k=0}^{2^{6}-1}E_{{\tilde{w}}_{k}}\left\{\log_{2}\sum_{i=0}^{2^{6}-1}\exp\left(-\frac{|a^{k}+{\tilde{w}}_{k}-a^{i}{|}^{2}-{\left|{\tilde{w}}_{k}\right|}^{2}}{2\xi_{k}^{2}/\alpha^{2}}\right)\right\}\right]}{2\;\left[2{\mathrm{FOM}}_{W}\cdot 4W\cdot 2^{b}\;+\;MP_{\mathrm{PS}}\;+\;P_{\mathrm{RFC}}\;+\;P_{\mathrm{PA}}\;\right]+\;P_{t}\;+\;P_{\mathrm{BB}}\;+\;NP_{\mathrm{RIS}}\;}. \end{array}$$

We note here that if more RIS elements are utilized for interference mitigation, then the considered system may require more energy.

Meanwhile, we consider a scenario utilizing high resolution A/Ds at the BS as a baseline for the energy efficiency comparison, which can further reduce the intra-beam interference at the cost of a significantly higher power consumption. By adopting high resolution A/Ds ($b_{\mathrm{HR}}=11$ bits quantization) at the BS, we can exploit a zero-forcing (ZF) algorithm for the design of the baseband digital precoder, i.e., $W_{\mathrm{ZF}}=\bar{\beta }H_{\mathrm{eq}}{\left(H_{\mathrm{eq}}H_{\mathrm{eq}}^{H}\right)}^{-1}$, where $H_{\mathrm{eq}}=\left[H_{d}^{T}+H_{r}^{T}\Phi G^{T}\right]F_{\mathrm{RF}}$ are the equivalent CSIs at the BS with the designed analog beamformers and the coefficients of the RIS, while $\bar{\beta }=\sqrt{\frac{1}{\mathrm{tr}(W_{\mathrm{eq}}W_{\mathrm{eq}}^{H})}}$ is the transmission power normalization factor. Then, the energy efficiency of utilizing high-resolution A/Ds and ZF precoding can been expressed as

(29) $$\begin{array}{c} \eta_{\mathrm{BS},\mathrm{ZF}}=\frac{\sum_{k=1}^{2}{\bar{R}}_{\mathrm{ZF},k}}{2\;\left[{\mathrm{FOM}}_{W}\cdot 4W\cdot 2^{b_{\mathrm{HR}}}\;+\;MP_{\mathrm{PS}}\;+\;P_{\mathrm{RFC}}\;+\;P_{\mathrm{PA}}\;\right]+\;P_{t}\;+\;P_{\mathrm{BB}}\;+\;NP_{\mathrm{RIS}}}. \end{array}$$

## 5. Simulation Results

In this section, we evaluate our proposed scheme through simulations. In the following simulations, unless otherwise specified, the number of antennas equipped at the BS is M=16, the number of RF chains equipped at the BS is $N_{\mathrm{RF}}=2$, the number of users is K=2, the number of antennas *N* at the RIS ranges from 32 to 1024, the Rician K-factor is set as $\varsigma_{k}=10$, and the number of scattering paths is set as $N_{\mathrm{cl}}=10$. Meanwhile, two users are randomly located in the same beam-direction of the BS (the two users are separated from the perspective of the RIS) and the large scale propagation losses of two users have been compensated. The power consumption of *b*-bit quantization resolution A/Ds is $P_{A/D}={\mathrm{FOM}}_{W}\cdot 4W\cdot 2^{b}$, where ${\mathrm{FOM}}_{W}=500\;\mathrm{fJ}/\mathrm{conversion}\text{-}\mathrm{step}$ is a typical value at 500 MHz [24]. The baseband signal processing power consumption is $P_{\mathrm{BB}}=200$ mW, the power consumption of the phase shifters is $P_{\mathrm{PS}}=10$ mW, and the power amplifier power consumption is $P_{\mathrm{PA}}=10\times M$ mW [15,24,28]. We note here that the power consumption of the each RIS element is assumed to be $P_{\mathrm{RIS}}=10$ mW.

**Figure 2:**
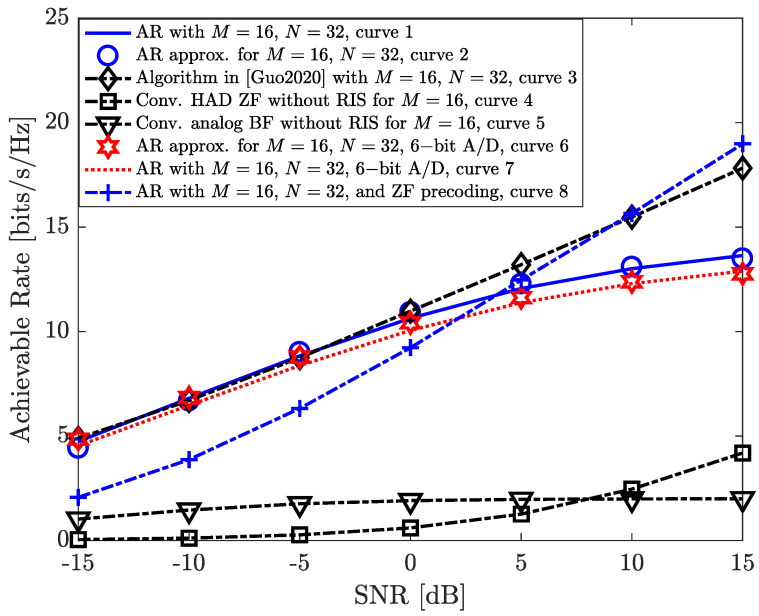
Average achievable sum−rate (bits/s/Hz) versus receive SNR (dB) performance comparison with M=16 for different benchmark schemes [9].

In Figure 2, we illustrate the achievable sum-rate of the considered scenario versus the pre-beamforming receive SNR. Here, the pre-beamforming SNR is defined as $\mathrm{SNR}=\frac{E_{s}}{\sigma^{2}}$. First, we compare the derived closed-form approximation in Equation (22) of Theorem 1 with the achievable sum-rate performance obtained from simulations (curves 1 and 2). In addition, we compare the obtained achievable rate results with different benchmark schemes, i.e., the weighted sum-rate maximization algorithm with RIS-assisted fully-digital MIMO system [9] (curve 3), the HAD architecture adopting conventional zero-forcing precoding without the RIS [6] (curve 4), and the HAD architecture utilizing only maximum ratio transmit (MRT) analog beamforming without the RIS (curve 5). In addition, compared with conventional schemes with HAD architecture without the RIS (curves 4∼5), the achievable sum-rate of the proposed algorithm with RIS-assisted HAD architecture (curve 1) can reach a significantly higher achievable sum-rate performance, which demonstrates the effectiveness of our proposed scheme for intra-beam interference mitigation. Interestingly, the rate performance gap between the proposed algorithm (curve 1) and the weighted sum-rate maximization algorithm with fully digital systems (curve 3) is marginal in low-to-medium SNR regimes, e.g., −15∼5 dB. Meanwhile, we present the derived closed-form approximation and the simulation results for the rate performance of our proposed scheme with six-bit PAM signals as well as six-bit A/Ds equipped at the BS (curves 6 and 7), which is provided in Equation (24). It is interesting to note here that the rate performance degradation between curves 1 and 7 is marginal.

**Figure 3:**
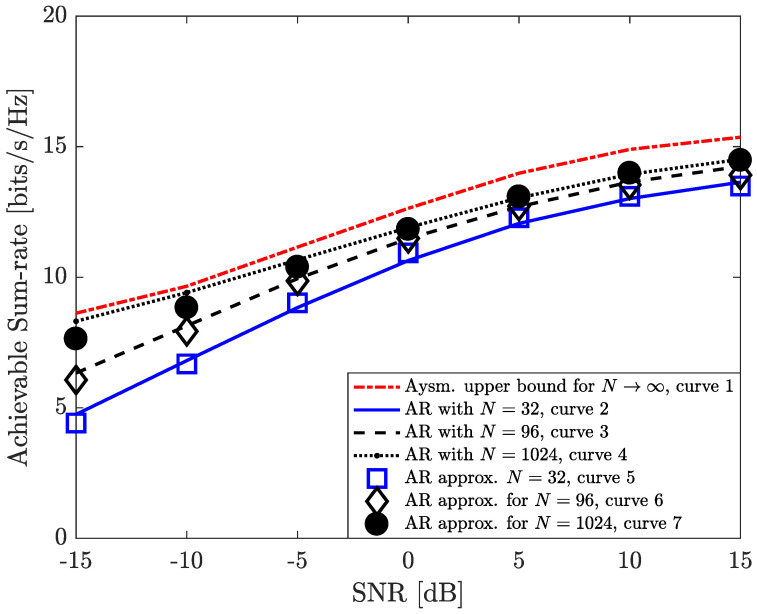
Asymptotic achievable sum−rate upper bound for the considered scenario in the regime with a large number of RIS elements.

In Figure 3, we further illustrate the asymptotic upper bound of the achievable sum-rate performance of our proposed algorithm in a regime with a large number of RIS elements (curve 1). In addition, we verify the correctness of Equation (23) in Corollary 1 for different numbers of RIS elements N∈{32,96,1024} (curves 2∼4). Our simulation results show that a larger number of RIS elements can provide a higher passive beamforming gain to improve the achievable sum-rate performance. On the other side, the involvement of more RIS elements to reach the considered interference mitigation may lead to higher energy consumption on the part of the considered system. Because the rate performance gaps are marginal in the medium-to-high SNR regime, e.g., 5∼15 dB (curves 2∼4), it would be interesting to determine the most reasonable number of RIS elements for mitigating intra-beam interference in terms of energy efficiency.

**Figure 4:**
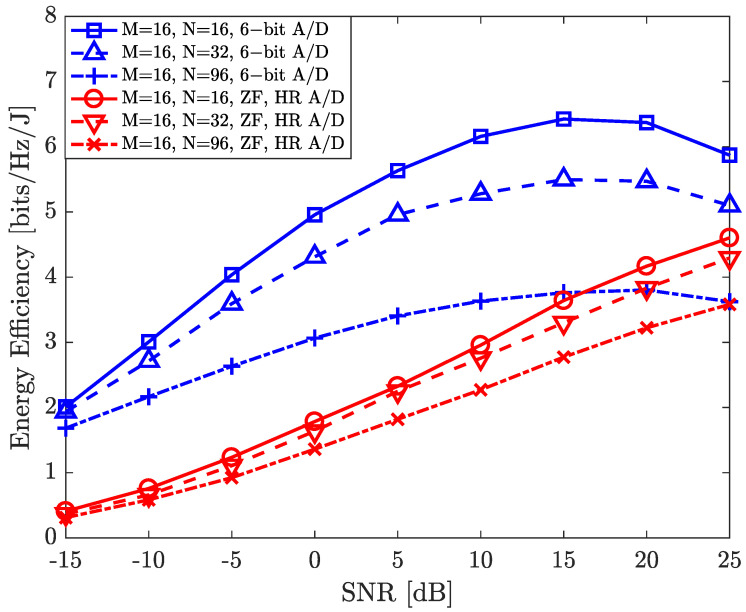
An illustration of the proposed hybrid architecture with a WPA and low−resolution A/Ds equipped at the BS and users for data transmission.

In Figure 4, we illustrate three sets of energy efficiency comparisons. Simulation results show that our proposed algorithm with six-bit A/D and six-bit PAM signals can have significantly higher energy efficiency compared to the scenario with high-resolution A/Ds and ZF baseband digital precoding. Meanwhile, the achievable rate of the proposed algorithm with high-resolution A/Ds and ZF baseband digital precoding is illustrated in Figure 2 (curve 8). We note here that the total power consumption includes the power consumption required to activate the RIS elements. Our simulation results demonstrate that the maximum energy efficiency of the proposed algorithm with the six-bit A/D is 6.2 bits/Hz/J for N=16, while the maximum energy efficiency of the proposed algorithm with high-resolution A/Ds (11-bit) and ZF baseband digital precoding is 4.2 bits/Hz/J. This confirms the superiority of the proposed algorithm in terms of energy efficiency. In addition, involving a larger number of RIS elements for interference mitigation can improve the achievable sum-rate performance. At the same time, the total energy consumption of the system increases with an increasing number of RIS elements. Simulation results illustrate that our proposed algorithm can provide reasonable intra-beam interference mitigation performance with significantly high energy efficiency by exploiting a relatively small number of RIS elements, e.g., N=16.

## 6. Conclusions

In this paper, we have proposed an innovative analog-only beamforming-based intra-beam inter-user interference mitigation algorithm for the downlink transmission of RIS-assisted HAD mmWave systems equipped with low-resolution A/Ds. We analytically obtain the closed-form approximation expressions for the achievable rate performance and establish a stringent upper bound. Utilizing low-resolution A/Ds at the BS allows the considered RIS-assisted HAD mmWave system to function with a relatively high energy efficiency for the downlink transmission, which we evaluated via simulations and compared to various benchmark schemes. Our analytical and simulation results prove that joint design of the analog beamforming coefficients at the BS and the RIS can effectively mitigate the intra-beam inter-user interference without causing extra energy consumption, even with a relatively small number of RIS elements.

## Figures and Tables

**Table 1:** Comparison between previous related works.

References	mmWave Frequency	Architecture	RIS-Assisted	Interference Mitigation/Rate Maximization	System Complexity	Low-Resolution A/Ds	Energy Efficiency
Work [9]	No	Fullydigital	✓	✓	High	No	Low
Work [12]	✓	Fullydigital	✓	✓	High	No	Low
Work [13]	No	Fullydigital and Fullduplex	✓	✓	Veryhigh	No	Low
Work [16]	✓	HAD	✓	No	Medium	No	Medium
Work [20]	✓	Fullydigital	✓	No	High	✓	Medium
Work [18]	✓	Fullydigital	✓	✓	High	✓	Medium
Work [19]	No	Fullydigital	✓	✓	High	✓	Medium
Work [21]	No	Fullydigital	✓	No	High	No	Low
Work [23]	No	Fullydigital	✓	✓	High	No	Low
Proposed algorithm	✓	HAD	✓	✓	Medium	✓	High

## Data Availability

Data are contained within the article.

## Appendix A

The result follows by substituting Equations (12), (13), (17), (18), (20) and (21) into (10). By omitting some negligibly small parts which neither dominate the performance nor scale up with *N* and *M*, we can obtain closed-form approximation expressions after some mathematical manipulation. With the designed power allocation coefficient for user 1, denoted as $\rho_{1}$, the analog beamformers at the BS $f_{L,1}$ and $f_{R,1}$, and the designed phase coefficients $\Phi ={\left(h_{r,1}^{T}⊙h_{N}^{H}\right)}^{H}e^{j\pi }$, the the received signal at user 1 can be expressed as

(A1) $$\begin{array}{c} y_{1}=\left[h_{d,1}^{T}+h_{r,1}^{T}\Phi G^{T}\right]\left(\sqrt{\rho_{1}}f_{L,1}+\sqrt{1-\rho_{1}}f_{R,1}\right). \end{array}$$

Then, the receive energy of the signal is $D_{1}=E\left[y_{1}^{2}\right]$, which can be approximated by

(A2) $$\begin{array}{cc} D_{1}\approx & \frac{\varsigma_{d}}{\varsigma_{d}+1}\rho_{1}M-2\rho_{1}\sqrt{N}R\left[h_{d,L,1}^{T}h_{M}^{*}\right]+2\sqrt{\rho_{1}\left(1-\rho_{1}\right)}N\left[R\left[h_{M}^{T}h_{d,L,1}^{*}\frac{h_{r,1}^{T}h_{r,2}^{*}}{|h_{r,1}^{T}h_{r,2}^{*}|}\right]\right]\\ \; & +\left(1-\rho_{1}\right)MN-\frac{M}{\sqrt{N}}R\left[\frac{h_{r,2}^{T}h_{r,1}^{*}}{|h_{r,1}^{T}h_{r,2}^{*}|}\right]. \end{array}$$

Meanwhile, with the predesigned analog beamformers, the interference signal received at user 1 can be approximated by

(A3) $$\begin{array}{cc} I_{1}\approx & \frac{\rho_{2}}{\left(\varsigma_{d}\;+\;1\right)M}{\left|h_{d,S,1}^{T}h_{M}^{*}\right|}^{2}\;+\;\frac{\left(1\;-\;\rho_{2}\right)}{M}{\left|h_{d,L,2}^{T}h_{M}^{*}\right|}^{2}\\ \; & -\;\frac{2\sqrt{\rho_{2}\left(1\;-\;\rho_{2}\right)\;N}}{M}\;{\left[R\left(h_{d,L,1}^{T}h_{M}^{*}\right)\right]}^{2}+\;\frac{\rho_{2}N}{M}\;{\left|h_{M}^{T}h_{d,L,2}^{*}\right|}^{2}\;. \end{array}$$

Similarly, the signal received at user 2 can be expressed as

(A4) $$\begin{array}{c} y_{2}=\left[h_{d,2}^{T}+h_{r,2}^{T}\Phi G^{T}\right]\left(\sqrt{\rho_{2}}f_{L,2}+\sqrt{1-\rho_{2}}f_{R,2}\right). \end{array}$$

Thus, we can obtain the energy of the signals received at user 2 as follows:

(A5) $$\begin{array}{cc} D_{2}\approx & \frac{\varsigma_{d}}{\varsigma_{d}+1}\rho_{2}M+\;\frac{M\left(1\;-\;\rho_{2}\right)}{N}{\left|h_{r,2}^{T}h_{r,1}^{*}\right|}^{2}\\ \; & -\;\frac{2\sqrt{\rho_{2}\;\left(1\;-\;\rho_{2}\right)}M}{\sqrt{N}}R\left[h_{r,1}^{T}h_{r,2}^{*}\right]\;+\;2\sqrt{\rho_{2}\;\left(1\;-\;\rho_{2}\right)}R\left[h_{d,L,2}^{T}h_{M}^{*}\right]. \end{array}$$

In addition, the interference signals received at user 2 can be approximated by

(A6) $$\begin{array}{cc} I_{2}\approx & \frac{\rho_{1}}{\left(\varsigma_{d}\;+\;1\right)M}{\left|h_{d,S,2}^{T}h_{d,L,1}^{*}\right|}^{2}\;+\;\frac{1\;-\;\rho_{1}}{M}{\left|h_{d,L,1}^{T}h_{M}^{*}\right|}^{2}. \end{array}$$

This completes the proof.

## References

[1] Wong V.W.S., Schober R., Ng D.W.K., Wang L.C. (2017). Key Technologies for 5G Wireless Systems.

[2] Rappaport T.S., MacCartney G.R., Samimi M.K., Sun S. (2015). Wideband millimeter-wave propagation measurements and channel models for future wireless communication system design. IEEE Trans. Commun..

[3] Liu C., Li M., Hanly S.V., Whiting P., Collings I.B. (2018). Millimeter-wave small cells: Base station discovery, beam alignment, and system design challenges. IEEE Wirel. Commun..

[4] Andrews J., Buzzi S., Wan C., Hanly S., Soong A.L.A., Zhang J. (2014). What will 5G be?. IEEE J. Sel. Areas Commun..

[5] Heath R.W., Prelcic N.G., Rangan S., Roh W., Sayeed A.M. (2016). An overview of signal processing techniques for millimeter wave MIMO systems. IEEE J. Sel. Top. Signal Process..

[6] Zhao L., Ng D.W.K., Yuan J. (2017). Multi-user precoding and channel estimation for hybrid millimeter wave systems. IEEE J. Sel. Areas Commun..

[7] Ouamri M.A., Oteşteanu M.E., Isar A., Azni M. (2020). Coverage, handoff and cost optimization for 5G heterogeneous network. Phys. Commun..

[8] Wu Q., Zhang R. (2020). Towards smart and reconfigurable environment: Intelligent reflecting surface aided wireless network. IEEE Commun. Mag..

[9] Guo H., Liang Y.C., Chen J., Larsson E.G. (2020). Weighted sum-rate maximization for reconfigurable intelligent surface aided wireless networks. IEEE Trans. Wirel. Commun..

[10] Wu Q., Zhang R. (2019). Intelligent reflecting surface enhanced wireless network via joint active and passive beamforming. IEEE Trans. Wirel. Commun..

[11] Adam A.B.M., Ouamri M.A., Muthanna M.S.A., Li X., Elhassan M.A.M., Muthanna A. (2023). Real-time and security-aware precoding in RIS-empowered multi-user wireless networks. arXiv.

[12] Ye J., Kammoun A., Alouini M.S. (2022). Reconfigurable intelligent surface enabled interference nulling and signal power maximization in mmWave bands. IEEE Trans. Wirel. Commun..

[13] Saeidi M.A., Emadi M.J., Masoumi H., Mili M.R., Ng D.W.K., Krikidis I. (2021). Weighted sum-rate maximization for multi-IRS-assisted full-duplex systems with hardware impairments. IEEE Trans. Cogn. Commun. Netw..

[14] Alkhateeb A., Leus G., Heath R.W. (2015). Limited feedback hybrid precoding for multi-user millimeter wave systems. IEEE Trans. Wirel. Commun..

[15] Zhao L., Li M., Liu C., Hanly S.V., Collings I.B., Whiting P.A. (2020). Energy efficient hybrid beamforming for multi-user millimeter wave communication with low-resolution A/D at transceivers. IEEE J. Sel. Areas Commun..

[16] Pradhan C., Li A., Song L., Vucetic B., Li Y. (2020). Hybrid precoding design for reconfigurable intelligent surface aided mmWave communication systems. IEEE Wirel. Commun. Lett..

[17] Zhang W., Xu J., Xu W., Ng D.W.K., Sun H. (2021). Cascaded channel estimation for IRS-assisted mmWave multi-antenna with quantized beamforming. IEEE Commun. Lett..

[18] Xiu Y., Zhao J., Basar E., Renzo M.D., Sun W., Gui G., Wei N. (2021). Uplink achievable rate maximization for reconfigurable intelligent surface aided millimeter wave systems with resolution-adaptive ADCs. IEEE Wirel. Commun. Lett..

[19] Dai J., Wang Y., Pan C., Zhi K., Ren H., Wang K. (2021). Reconfigurable intelligent surface aided massive MIMO systems with low-resolution DACs. IEEE Commun. Lett..

[20] Zhi K., Pan C., Ren H., Wang K. (2020). Uplink achievable rate of intelligent reflecting surface-aided millimeter-wave communications with low-resolution ADC and phase noise. IEEE Wirel. Commun. Lett..

[21] Zhou S., Xu W., Wang K., Renzo M.D., Alouini M.S. (2020). Spectral and energy efficiency of IRS-assisted MISO communication with hardware impairments. IEEE Wirel. Commun. Lett..

[22] Pan C., Zhou G., Zhi K., Hong S., Wu T., Pan Y., Ren H., Renzo M.D., Swindlehurst A.L., Zhang R. (2022). An overview of signal processing techniques for RIS/IRS-aided wireless systems. IEEE J. Sel. Top. Signal Process..

[23] Zhong K., Hu J., Pan C., An D. (2022). RIS-Aided Multiple User Interference Mitigation via Fast Successive Upper-Bound Minimization Method. IEEE Commun. Lett..

[24] Mo J., Alkhateeb A., Abu-Surra S., Heath R.W. (2017). Hybrid architectures with few-bit ADC receivers: Achievable rates and energy-rate tradeoffs. IEEE Trans. Wirel. Commun..

[25] Zhang J.A., Liu F., Masouros C., Heath R.W., Feng Z., Zheng L., Petropulu A. (2021). An overview of signal processing techniques for joint communication and radar sensing. IEEE J. Sel. Top. Signal Process..

[26] Mo J., Heath R.W. (2018). Limited feedback in single and multi-user MIMO systems with finite-bit ADCs. IEEE Trans. Wirel. Commun..

[27] Rial R.M., Rusu C., Prelcic N.G., Alkhateeb A., Heath R.W. (2016). Hybrid MIMO architectures for millimeter wave communications: Phase shifters or switches?. IEEE Access.

[28] Zhang J., Dai L., Sun S., Wang Z. (2016). On the spectral efficiency of massive MIMO systems with low-resolution ADCs. IEEE Commun. Lett..

